# 

**Published:** 1915-01-02

**Authors:** 


### CONTENTS.

PAGE

**New Year's Day 1915** ... ... ... ... 299

**The Metropolitan Asylums Board and Permanent Invalid Children** ... ... ... 300

**Hospital and Institutional News** ... ... ... 301

The Voluntary Hospitals Brigade—Bombardment of the Hartlepools—Secretary Guarding German Prisoners—Metropolitan Hospital Sunday Fund—The Historian of the Royal Army Medical Corps—Should Medical Officers Dispense?—Scientific Pharmacy in Large Institutions—Ambulance Barges for Flanders—A Poor-Law Christmas Card—"King Albert's Book"—The Mechanism of Modern War—Death of Sir Robert M. Simon, M.D., F.R.C.P.—The St. John Ambulance Brigade Hospital—Tanneries and Infectious Diseases—The Hospitals and War Risks—Honour for a Hospital Secretary—Women Doctors and Infirmary Posts—A Scarcity of Medical Women—Teaching Hospitals and Pharmacological Classes—The New Year's Reference Books—The Sale of Laudanum—Drug Habits and the New Pharmacopœia—This Week's Drug Market.

**The Work of the R.A.M.C. in War** ... ... 307

Evacuation Difficulties: The Triumph of the Motor Ambulance.

**Ambulance Work, the Public, and the National Guard** ... ... ... ... ... ... 309

**Round the World** ... ... ... ... ... 310

Fellow-Workers and the Roll of Honour.

[*See over.*]

### CONTENTS—(*continued*).

PAGE

**Christmas in the Institutions** ... ... ... 311
Christmas at Addenbrooke's, Cambridge.
Great Northern Central Hospital.
Fulham Infirmary.
Wilkinson Sanatorium, Bolton.
Christmas in a Chinese Hospital.
Leeds Hospital for Women and Children.
Christmas in a Base Hospital.
The National Sanatorium, Benenden, Kent.
Cardiff.
The Cardiff Military Hospitals.
Gravesend Hospital : A Hostile Aeroplane.

**Hospitals and the War** ... ... ... ... 315
Cardiff—Leicester : A Christmas Convoy—Netley : The Welsh War Hospital—Norfolk and Norwich Hospital—Indians at Brighton—Middlesbrough : A Memory of the Scarborough Raid.

**The East Coast Hospitals and the Wounded...** 318

PAGE

**Metropolitan Hospital Sunday Fund** ... ... 317
Annual Meeting of Constituents.

**Buildings Contemplated** ... ... ... ... 318

**The Editor's Letter-Box** ... .. ... ... 320
Soldiers' Dental Aid Fund—Institutions for Sailors.

**Medical Appointments** ... ... ... ... 320

**Personalia** ... ... ... ... ... ... 320

**Obituary ...** ... ... ... ... ... ... 320

**Institutional Needs** ... ... ... ... ... 320

**The Institutional Worker** ... ... (Suppl.) 1
Answer to December Question : Contributions from Out-Patients.
Ventilation of the Operating Theatre.
Question for January.
Employment and Training.
The Sick and in Need.
The Housekeeper's Department.
The Institutional Worker and his Work.

